# Effect of TNF-α on human ARPE-19-secreted proteins

**Published:** 2008-12-11

**Authors:** Eunkyung An, Heather Gordish-Dressman, Yetrib Hathout

**Affiliations:** 1 Center for Genetic Medicine, Children’s National Medical Center, Washington, D.C; 2 Program in Biochemistry and Molecular Genetics, Institute of Biomedical Science, The George Washington University, Washington, D.C

## Abstract

**Purpose:**

To identify cytokine-induced changes in the secretome of human retinal pigment epithelial (RPE) cells and their potential implication in age-related macular degeneration pathogenesis.

**Methods:**

Stable isotope labeling by amino in cell culture (SILAC) was used in combination with liquid chromatography tandem mass spectrometry (LC-MS/MS) to measure differential protein secretion from tumor necrosis factor-α (TNF-α) treated ARPE-19 versus untreated ARPE-19 cells. Typically, one set of cells was subcultured in a medium in which Arg and Lys were replaced by ^13^C_6_-Arg and ^15^N_2,_ ^13^C_6_-Lys while the other set of cells was grown in unlabeled medium. The fully labeled cells were then treated with TNF-α, while unlabeled cells were left untreated. Spent media from both treated and untreated cells were collected, mixed at 1:1 ratio, and processed for LC-MS/MS analysis. Labeled and unlabeled peptide pairs were identified and their intensities were used to determine protein ratios in TNF-α treated cells versus untreated cells. To validate the data, we performed a reverse experiment in which unlabeled cells were treated with TNF-α while labeled cells were kept untreated.

**Results:**

A total of 146 proteins were identified as putatively secreted proteins in the spent medium of ARPE-19 cells and only six among these were differentially secreted following TNF-α treatment. Secretion of complement 3 and sulfhydryl oxidase-1 was increased by twofold, fibronectin by 1.7 fold, plasminogen activator inhibitor 1 by 1.9 fold and syndecan-4 by 4.35 fold while secretion of trans-golgi network protein-2 was decreased by twofold.

**Conclusions:**

TNF-α modulates secretion of specific proteins in ARPE-19 cells. These proteins are involved in pathways relevant to AMD pathogenesis (e.g., extracellular matrix remodeling, complement pathway, and angiogenesis).

## Introduction

Age-related macular degeneration (AMD) is a leading cause of blindness in elderly individuals [1]. AMD is characterized by extracellular deposits (e.g., drusen) that accumulate beneath the retinal pigment epithelium (RPE) and along Bruch’s membrane [2–4]. Previous studies have established a strong association between the Tyr402His variant in complement factor H (Y402H CFH) and the risk of developing AMD [5–8]. More recently, a variant in the promoter region of the gene encoding for the serine protease HtrA1 was found to be a second major risk factor for AMD [9–11]. However, the mechanisms by which these single point mutations contribute to AMD pathogenesis (e.g., drusen accumulation and choroidal neovascularization) are still not well understood.

RPE cells have long been suspected to be a source for at least some of the material that accumulates in drusen [2,12]. Our study of the secretome of human primary RPE cultures clearly demonstrated that these cells can express and secrete several proteins found in drusen including CFH and complement components [13]. RPE are highly specialized epithelial cells that maintain integrity of the blood-retina barrier while performing vital functions such as phagocytosis of the outer segment of photoreceptor cells, recycling of visual pigment, and transport of nutrients to the photoreceptors [14]. Hence, RPE cells are continuously exposed to oxidative stress and proinflammatory stimuli that may disrupt their extracellular environment and challenge their local homeostasis.

The presence of few small drusen in the macula of individuals over age 40 is normal and usually not harmful. However, an increase in number and size of these deposits is a strong indication of a progressive AMD. Previous studies have shown that drusen attract macrophages to the sub-RPE space [15,16]. Activated macrophages are known to produce tumor necrosis factor-α (TNF-α), a pleotropic cytokine, which has been shown to stimulate production of monocyte chemotactic protein (MCP-1) by RPE cells thus recruiting more macrophages to the vicinity of the sub-RPE space [17,18]. Chronic exposure of RPE cells to cytokine may alter their protein secretion pattern and increase the risk of complex deposition. However, RPE cells express both soluble and cell surface cytokine receptors as well as a variety of other factors, thus giving them some degree of control to modulate the effect of cytokines [19]. This regulation of the extracellular environment may be compromised in RPE cells carrying AMD genetic risk variants (e.g., the Y402H CFH and the HtrA1 promoter polymorphisms), leading to an accumulation of extracellular deposits.

In this preliminary study we decided to examine the overall effect of TNF-α on the regulation of protein secretion by RPE cells and bring insight into cytokine-induced alterations and their role in AMD pathogenesis. We used ARPE-19 cells as a model to optimize the conditions for future studies using human primary RPE cell culture.

Stable isotope labeling by amino acid in cell culture (SILAC) is a simple and accurate strategy for proteome profiling in cell culture systems [20]. Basically, one set of cells are grown in a medium where some of the amino acids, usually arginine (Arg) as well as lysine (Lys), are labeled with stable isotopes (e.g., ^13^C and/or ^15^N), while the comparative sample is grown in medium containing unlabeled amino acids. This approach is the most systematic way to uniformly label all the proteins within the cell and obtain accurate protein ratios between two samples. Indeed, variations usually due to experimental handling are minimized in this case since labeled and unlabeled cells can be mixed before subcellular fractionation and protein extraction. For these reasons, the method has gained tremendous popularity and has been successfully implemented to study cell signaling [21–24], phosphoproteomics [25–27], and cell-secreted proteins [13,28,29].

In this study, we implemented the SILAC strategy to define differential protein secretion in human RPE cell line (ARPE-19) exposed to a non-lethal dose of TNF-α. Analysis of these proteins should bring insight into the response of RPE cells to cytokines and eventual implication in AMD pathogenesis.

## Methods

### SILAC and TNF-α treatment

**Figure 1:**
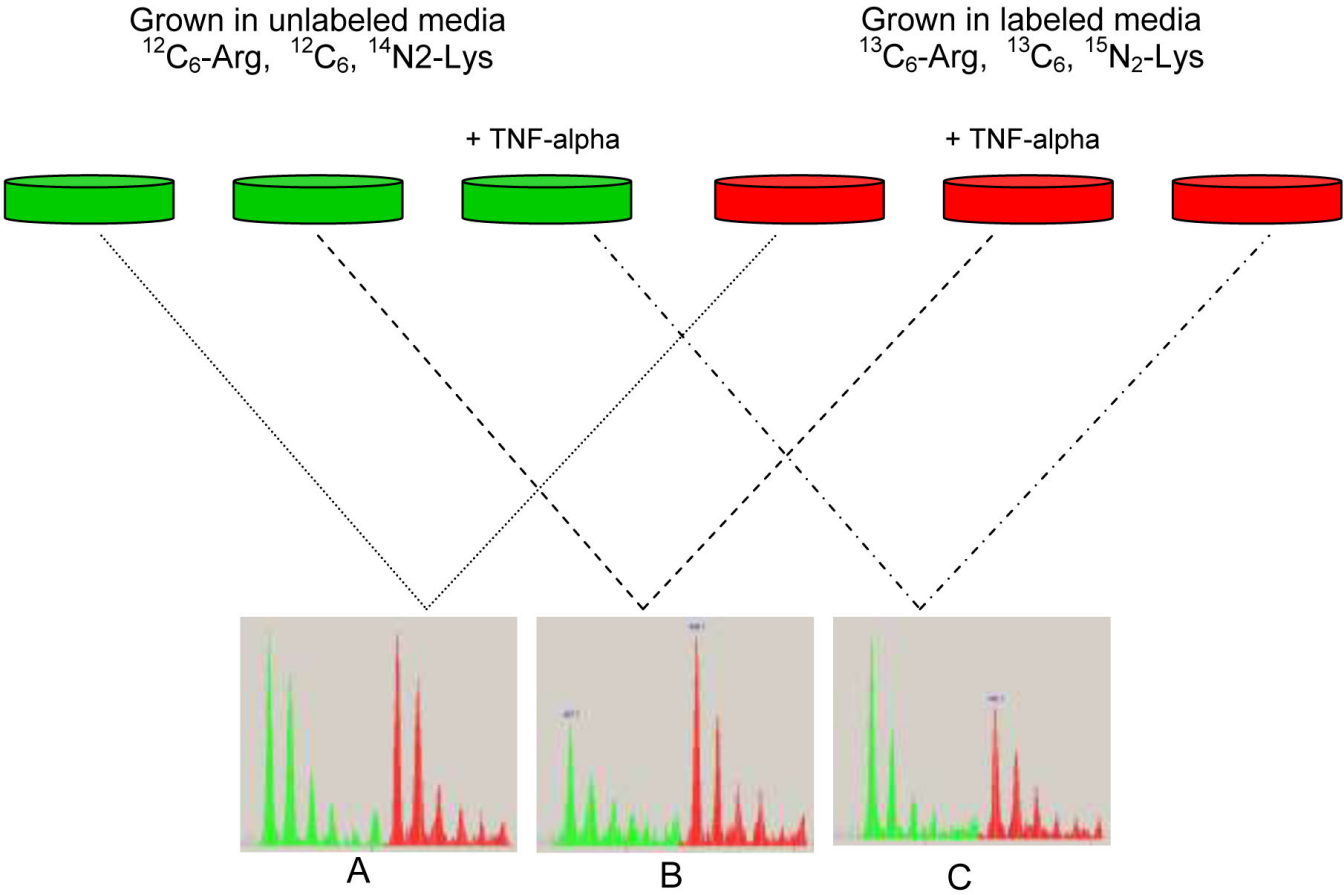
Experimental design to study the effect of TNF-α on ARPE-19 secreted proteins. ARPE-19 cells were grown in medium where Lys and Arg were replaced by ^15^N, ^13^C_6_-Lys, and ^13^C_6_-Arg. In parallel, ARPE-19 cells were cultured in medium with normal Lys and Arg. After cells were fully labeled with heavy and light amino acids, 10 ng/ml of TNF-α was added into each flask of labeled cells and unlabeled cells and incubated for 24 h in serum-free medium while the other remaining flasks were incubated in the same serum-free medium without TNF-α. **A**: For the control experiment, spent medium from labeled cells (e.g., untreated) was mixed at a 1:1 ratio with spent medium from unlabeled cells (e.g., untreated). **B**: To check the effect of TNF-α on ARPE−19 secreted proteins, we mixed the spent medium from labeled cells (e.g., treated with TNF-α) at a 1:1 ratio with spent medium from unlabeled cells (untreated). **C**: To validate the data, we performed a reverse experiment in which spent medium of labeled cell (e.g., untreated) was mixed at a 1:1 ratio with unlabeled cells (e.g., treated with TNF-α). Sample pairs were then processed as described in the method for subsequent LC-MS analysis and determination of protein ratios.

ARPE-19 was purchased from the American Type Culture Collection (ATCC; Manassas, VA). The cells were routinely maintained in Dulbecco’s modified Eagle’s medium and Ham’s F12 medium (DMEM/F12) containing 10% FBS (ATCC), 100 U/ml of penicillin, 100 µg/ml of streptomycin (Invitrogen, Gibco, Carlsbad, CA), and 1 mM of sodium pyruvate (Sigma Aldrich, St. Louis, MO). To study the effect of TNF-α on ARPE-19 secreted proteins, we performed differential stable isotope labeling of amino acids in cell culture and used mass spectroscopy to measure ratios of individual secreted proteins in TNF-α treated ARPE-19 and untreated ARPE-19 cells (Figure 1). One set of ARPE-19 cells were subcultured in Dulbecco’s modified Eagle’s medium and Ham’s F12 medium (DMEM/F12; Atlanta Biologicals, Lawrenceville, GA) custom-made medium where Arg and Lys were replaced by ^13^C_6_-Arg and ^15^N_2,_ ^13^C_6_-Lys. After at least 12 cell doublings (roughly four passages), cellular proteins were uniformly labeled with these stable isotope-containing amino acids. In parallel, the same numbers of cells were grown in DMEM/F12 unlabeled medium. Ten days after confluence, both labeled and unlabeled cells were washed 6 times with sterile PBS (Invitrogen, Carlsbad, CA) before incubating them in serum-free medium containing stable isotope-labeled amino acids for labeled cells and regular amino acids for unlabeled cells. The metabolically labeled cells were treated with 10 ng/ml TNF-α (a nonlethal dose), while unlabeled cells were kept untreated. After 24 h incubation, spent media of both treated and untreated cells were collected, mixed at 1:1 ratio and analyzed as described below. To validate the data, we performed a reverse experiment in which unlabeled cells were treated with TNF-α while labeled cells were kept untreated.

### Analysis of ARPE-19-secreted proteins

Spent media from TNF-α-treated and untreated ARPE-19 cells were collected and filtered through 0.2 µm polyethersulfone syringe filter (Corning Inc., Corning, NY) to remove floating cells and debris. Filtered media from treated and untreated cells were then mixed at 1:1 ratio (v/v) and concentrated to dryness using centrifugal filtration (Millipore Corporation, Billerica, MA) followed by vacuum centrifugation. The resultant residue was then dissolved in 6 µl of Laemmli buffer containing 50 mM DTT and further fractionated by SDS–PAGE using 10% Tris HCl pre-cast criterion gels (Bio-Rad, Hercules, CA). The gel was then stained with Bio-Safe Coommassie (Bio-Rad) and each lane was excised into 36 to 43 bands. Each band was then in-gel digested using trypsin (Promega, Madison, WI), and the resultant peptides were extracted and analyzed by nano-LC-MS/MS as described in the next section.

### Preparation of microsomal fraction

After collection of spent media, treated and untreated cells were washed with PBS, dissociated in 0.05% trypsin-EDTA solution (Invitrogen) and transferred into previously weighed Eppendorf tube. Cells were pelleted by centrifugation at 300x g then washed with 2 volumes of PBS solution and 100 mM NaCl. Treated and untreated cells were then mixed at 1:1 ratio (w/w) and resuspended in 1.5 ml of Tris-EDTA mix buffer that contained 10 mM Tris HCl pH 7.4, 1 mM EDTA, 2.5 M sucrose (Sigma Aldrich), and 1 protease inhibitor tablet (Pierce, Rockford, IL) per 10 ml. The cells were then mechanically lysed by passing them 15 times through a 1 ml syringe with a 23 gauge needle. The suspension was centrifuged for 10 min at 4000x g at 4 °C, and the supernatant containing the microsomal fraction was transferred to a clean 1.5 ml Eppendorf tube and further centrifuged at 13,000x g for 20 min to obtain the microsomal pellet. The microsomal pellet was then resuspended in protein extraction buffer containing 7 M urea,2 M thiourea, 2% CHAPS (w/v), and 50 mM DTT. Protein concentration of each fraction was determined by Bio-Rad protein assay (Bio-Rad). Aliquots of 50 µg were separated on SDS–PAGE. Gel bands were excised, digested with trypsin, and the resultant peptides analyzed by liquid chromatography mass spectrometry as described in the next section.

### Nanoflow LC-MS/MS

Nano-LC tandem mass spectrometry was performed on an LC-packing system (Dionex Ultimate Capillary/Nano LC system, Dionex Corp., Sunnyvale, CA) connected to an a linear trap quadrupole (LTQ) instrument (Thermo Fisher Scientific, San Jose, CA). Each sample was injected via an auto-sampler and loaded onto a C18 trap column (5 μm, 300 μm i.d. x 5 mm; Dionex LC-Packings, Sunnyvale, CA) and washed with water containing 0.1% trifluoroacetic acid for 6 min at a flow rate of 10 μl/min. The sample was subsequently separated by a C18 reverse-phase column (3.5 μm, 100 μm x 15 cm, Agilent, Santa Clara, CA) at a flow rate of 200 nl/min. The mobile phases consisted of water with 0.1% formic acid (A) and 90% acetonitrile with 0.1% formic acid (B). A linear gradient from 5 to 65% of solvent B was employed over a period of 100 min to separate peptide mixture. Eluted peptides were introduced into the mass spectrometer via a 10 μm silica tip (New Objective Inc., Ringoes, NJ) adapted to a nano-electrospray source (Thermo Fisher Scientific Inc., Waltham, MA). The spray voltage was set at 1.7 kV and the heated capillary at 180 °C. LTQ was operated in the data-dependent mode in which each cycle consisted of one full-MS survey and subsequently three sequential pairs of intercalated zoom scans and MS/MS events. The targeted ions count in the mass spectrometer trap was 30,000 for full-MS scans, 3,000 for zoom scans, and 10,000 for MS/MS scans. Peptides were fragmented in the linear ion trap using collision-induced dissociation with the collision gas (helium) pressure set at 1.3 ml and the normalized collision energy value set at 35%. The zoom scan events, of higher resolution, were used to determine the charge state of each ion as well as the ratio of labeled to unlabeled peptide pairs using the ZoomQuant software developed by Halligan et al. [30].

### Database search

Protein identification was performed by searching the 17,806 human protein sequences in the SwissProt database (UniProtKB/Swiss-Prot release 54.6 of December 2007) using Bioworks 3.3 (Thermo Fisher Scientific, San Jose, CA) and indexed with assumptions for enzymatic tryptic hydrolysis with the possibility of two missed cleavages and the following modifications: 16 Da shift for oxidized Met, 6 and 8 Da shifts for incorporation of stable isotope labeled Arg and Lys, respectively. Stringent filtering criteria were used for database search and peptide identifications: DeltaCn (ΔCn) > 0.1, a variable threshold of *Xcorr* versus charge state (*Xcorr*=1.9 for z=1, *Xcorr*=2.5 for z=2, and *Xcorr*=3.5 for z=3), peptide probability-based score with a p value <0.001, and more than 2 different peptides per protein. A reverse database search using the same criteria gave a false positive rate of 0%, thus increasing confidence in protein identification:

$$\text{False Positive Rate = 2 x}\left(\frac{\text{Reverse Database Protein}}{\text{Reverse + Forward Database Protein}}\right)\text{ x 100}$$

### ZoomQuant analysis

For quantitative analysis we used ZoomQuant Software [30]. In this case proteins were identified using BioWorks 3.1 software that is compatible with ZoomQuant software to generate Sequest output files and extract zoom scans from the mass spectrometry raw data. Each peptide was manually validated by examining zoom scans to verify the labeled and unlabeled pairs. Identified peptides were required to have a matched peptide partner 6 Da greater for Arg terminating peptides, 8 Da greater for Lys containing peptides, and other proper combinations for missed cleavage peptides. Because manually checking zoom scan with pairs of labeled and unlabeled peaks is itself stringent, filtration based on the *X corr* was lowered in this case to increase proteome coverage (e.g., *X corr* ≥ 1.8 for doubly charged ions and *X corr* ≥ 2.5 for triply charged ions).

### Statistical analysis

To check the significance of differentially expressed proteins in TNF-α treated cells versus non-treated cells a *t*-tests for equal variances was performed using the Sidak method [31]. Basically an average ratio was calculated from the total scans of labeled and unlabeled peptide pairs detected for a given protein. These averaged ratios were then compared in the control pairs versus TNF-α treated cells and untreated cells in both the forward and reverse SILAC experiment. The obtained p values were then adjusted for the three way multiple comparisons using the Sidak method.

### Western blot analysis

To validate proteome profiling data, we cultured ARPE-19 cells in 10 cm dishes (Corning Inc. Life Sciences, Lowell, MA) to 100% confluence. Cells were washed 6 times with PBS to remove any residual FBS proteins, and treated with various TNF-α concentrations (0.25 to 20 ng/ml) in serum free media for 24 h. Spent media were collected and filtered though 0.2 µm Polyethersulfone syringe filter (Corning Inc.). To check NF-κB activation, we collected cell pellets, washed them with PBS, and lysed with 100 µl of RIPA (Sigma Aldrich, St. Louis, MO) buffer. Protein extracts were separated by SDS–PAGE on a 4 to 15% Tris HCl gradient gel (BioRad) under reducing conditions then transferred to a nitrocellulose membrane (Amersham Biosciences, Piscataway, NJ) for western blot analysis. Nonspecific binding was blocked with 5% milk (BioRad Laboratories Inc., Hercules, CA), and the membrane was incubated overnight with goat polyclonal antibody against human C3 (Complement Technology, Tyler, TX), rabbit polyclonal antibody against human IκB-α (Cell Signaling, Danvers, MA) or mouse monoclonal antibody against human vinculin (Sigma) in 2.5% milk. The next day, the membrane was rinsed with Tris buffer containing salt and Tween (TBST) and incubated with antigoat antibody (Jackson Lab, West Grove, PA) for C3, and antirabbit IgG (BioRad) for IκB-α or antimouse antibody (GE Healthcare, Buckinghamshire, England) for vinculin. All secondary antibodies were conjugated with horseradish peroxidase, and immunoblots were visualized by chemiluminescence (Amersham Biosciences Inc., Piscataway, NJ)

## Results

### Effect of TNF-α on the viability of ARPE-19 cells

**Figure 2:**
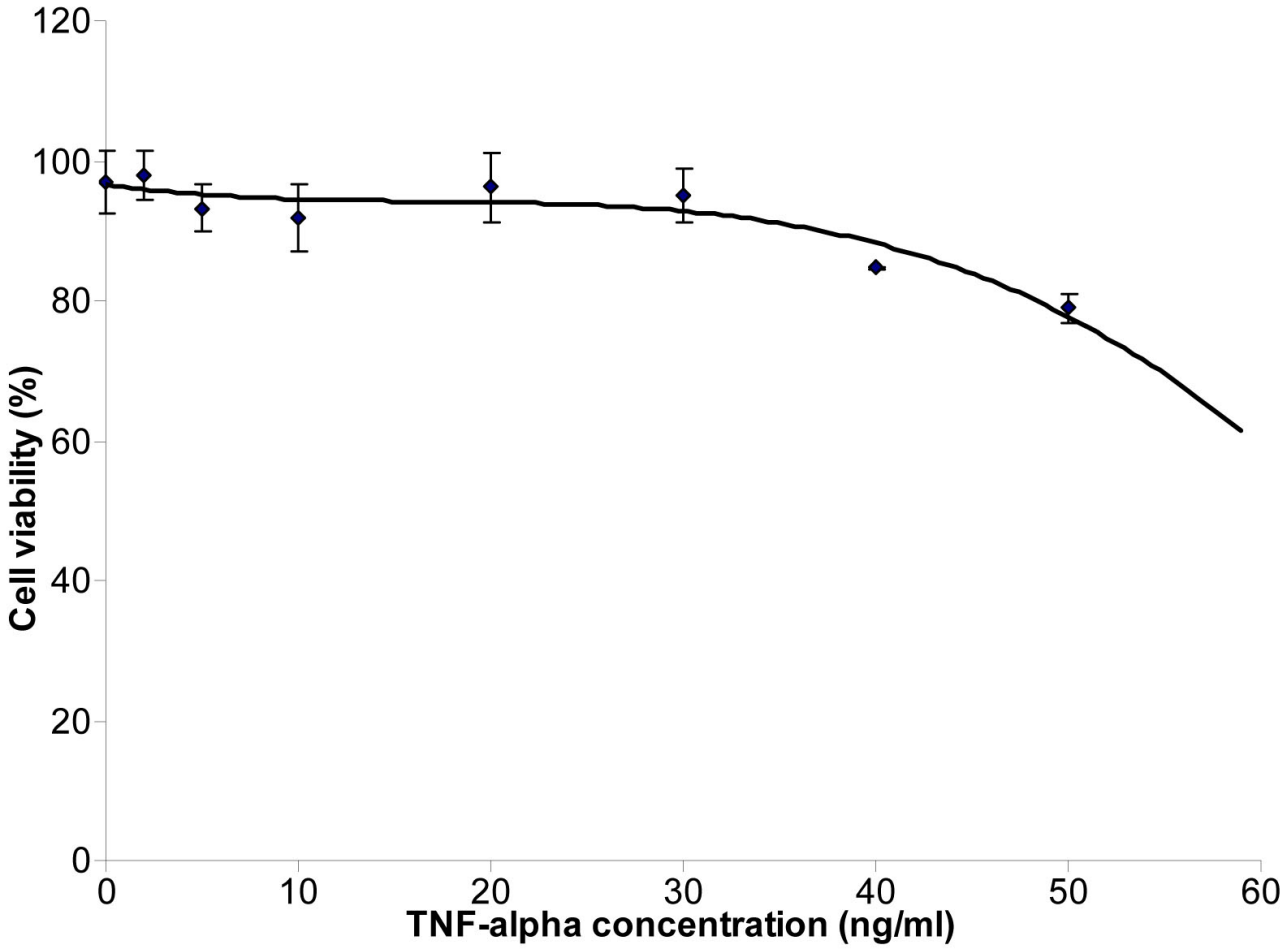
Cell viability in function of increased TNF-α concentration. Both viable and dead cells were counted using a hemocytometer. A percent of viable cells to the total cells was then calculated for each culture flask. Error bars represent standard deviations obtained from triplicate experiment for each dose of TNF-α used. To assess the number of cell death in the control group we have chosen not to normalize the values to 0 ng/ml of TNF-α.

To ensure minimum cell death and minimum release of intracellular proteins into the condition medium of TNF-α-treated ARPE-19 cells, we monitored cell viability after incubating them for 24 h with different concentrations of TNF-α using trypan blue exclusion. Figure 2 shows the percent of viable cells after 24 h incubation with different TNF-α concentrations. TNF-α concentration up to 30 ng/ml produced only a small percent of cell death (e.g., <10% compared to untreated cells). This data are in agreement with a previous study where it has been shown that RPE cells are more resistant to TNF-α-induced apoptosis than other cell type [32]. We have thus chosen 10 ng/ml as the optimal concentration to study the effect of TNF-α on ARPE-19-secreted proteins. This concentration resulted in minimum cell death (<5%) and detectable cell response.

### Effect of TNF-α on ARPE-19-secreted proteins

**Figure 3:**
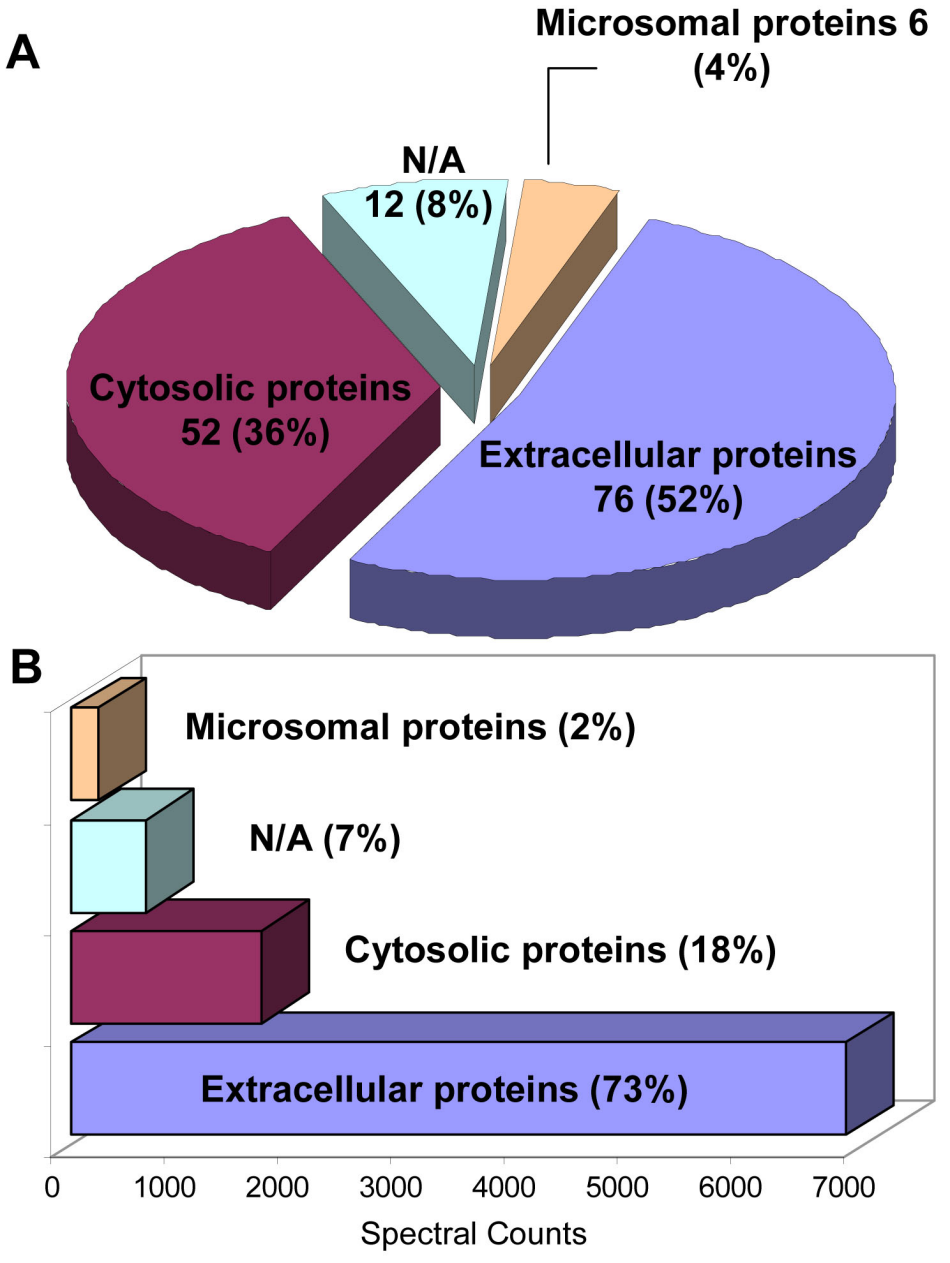
Cellular localization of proteins identified in spent medium of ARPE-19 cells. Panel **A** shows a pie chart depicting the subcellular distribution of the proteins identified in the spent medium of ARPE-19 cells. Panel **B** shows a bar graph plot representing the abundance of each class of proteins based on the spectral count of the total peptides detected. Other class of proteins with no known subcellular localization and no known function was annotated as (N/A) meaning not assigned.

One of the advantages of using the SILAC strategy is that all cellular proteins, including secreted proteins, are uniformly labeled with the stable isotope-labeled amino acids (e.g., ^13^C_6_-Arg, ^13^C_6_, ^15^N_2_-Lys). Thus, in a 1:1 mixture of spent media collected from labeled and unlabeled cells, most of the peptides generated from cellular proteins would be detected as pairs of labeled and unlabeled peaks, while peptides from residual bovine serum proteins and contaminant proteins, such as keratin, would be detected as single peaks. In this study, a total of 221 proteins were identified in the spent medium of ARPE-19 cells, but only 146 proteins were of RPE cell origin; these are listed in Appendix 1 with their accession number, peptide count, subcellular localization, and function. The cellular localization of these proteins as well as their function was deduced from their annotations in the UniProt database using the Protein Information Resources (PIR) tool. As shown in Figure 3A, 76 (52%) of these 146 proteins were found to be putatively secreted, 6 (4%) were of microsomal origin, 52 (36%) were of cytosolic origin, and the remaining 12 (8%) were unassigned. While the number of identified cytosolic proteins was high, their actual relative quantities estimated from spectral count were low compared to the secreted proteins (Figure 3B). All proteins in Appendix 1 were identified with at least two different peptides and validated by detection of labeled and unlabeled peptide pairs. Additional proteins that were identified by a single peptide pair in the spent medium of ARPE-19 cells are reported in Appendix 2. Even though each of these proteins was represented by a single peptide sequence, their identity was still validated by detection and sequencing of both labeled and unlabeled peptide pair. Detection of only one representative peptide for each of these proteins suggests that they might be present in very low abundance in ARPE-19 spent medium.

**Figure 4:**
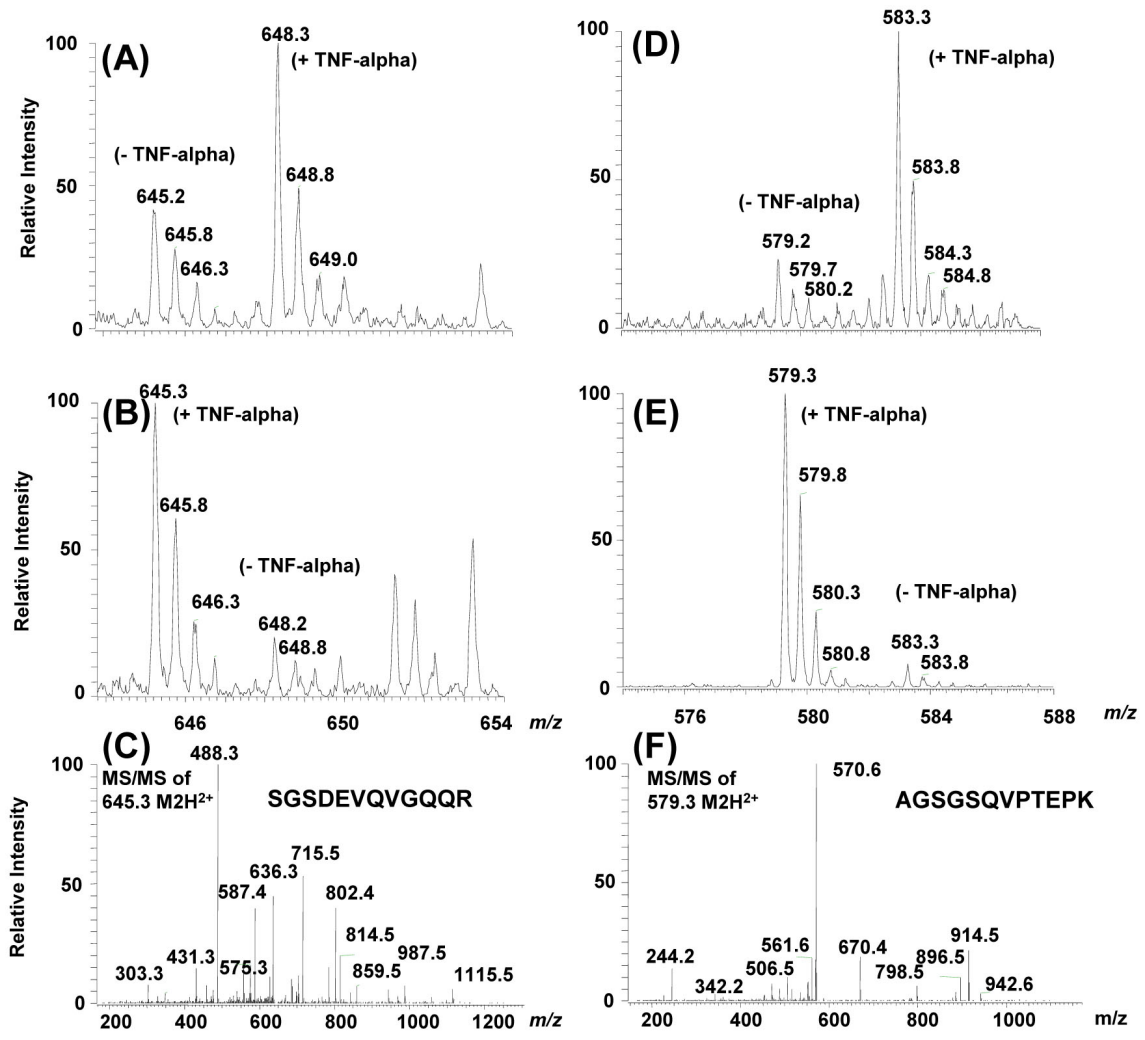
Zoom scan mass spectra of labeled and unlabeled peptide pairs detected for complement C3 and syndecan-4 proteins. Panels **A** and **D** show the mass spectra and the relative intensity of labeled and unlabeled peptide pairs detected for complement C3 (**A**) and for syndecan-4 (**D**) in TNF-α-treated ARPE-19 cells versus untreated cells. In this case labeled cells were treated with TNF-α (+TNF-α) while unlabeled cells remained untreated (-TNF-α). Panels **B** and **E** show the mass spectra obtained for these same set of peptides in the reverse experiment where unlabeled cells were treated with TNF-α (+TNF-α) while labeled cells remained untreated (-TNF-α). Panel **C** shows the tandem mass spectrum and the fragment ions obtained for the doubly-charged ion m/z=645.2 and confirming the peptide sequence [SGSDEVQVGQQR] of complement C3 protein. Panel **F** shows the tandem mass spectrum and the fragment ions obtained for the doubly-charged ions m/z=579.3 and confirming the peptide sequence [AGSGSQVPTEPK] of syndecan-4 protein.

**Figure 5:**
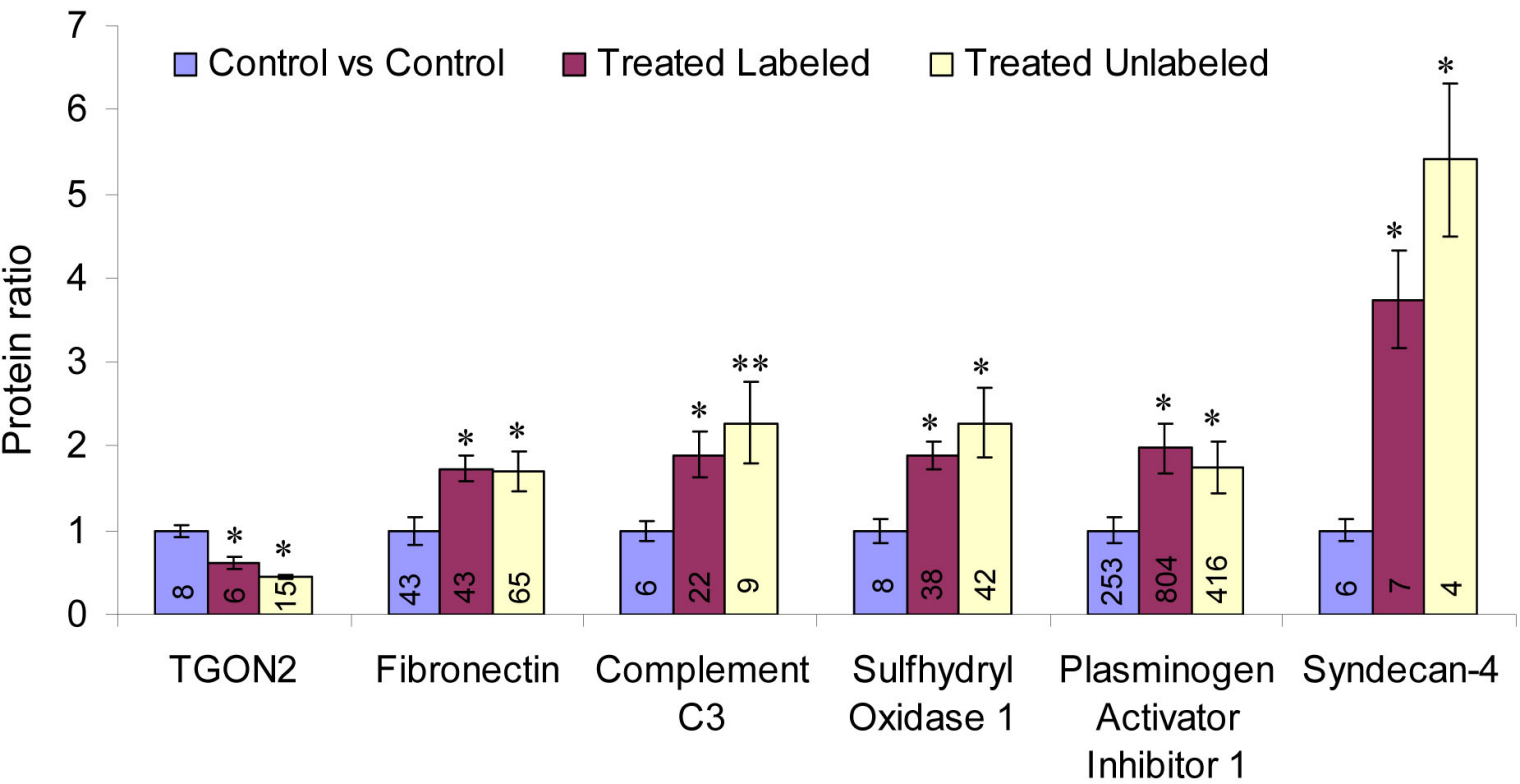
Bar graph for the 6 proteins whose secretion was found differentially altered in TNF-α-treated ARPE-19 cells. Blue bars indicate ratios in control versus control cells (both labeled and unlabeled cells were untreated). Purple bars indicate ratios in TNF-α-treated cells versus untreated cells and in this case SILAC labeled cells were treated with TNF-α while unlabeled cells were kept untreated. Yellow bars indicate ratios in TNF-α-treated cells versus untreated cells and in this case unlabeled cells were treated with TNF-α while SILAC-labeled cells were kept untreated. Numbers inside of bars represent the total MS scans of labeled and unlabeled peptide pairs detected for each protein. The error bars represent the standard deviation between ratios of these individual scans. The p-values were obtained using three pair-wise *t*-tests (Sidak method) where all ratios for a given protein in control versus control were compared to ratios obtained in TNF-α treated versus untreated cells in both the reverse and forward experiments and these values are as follow (* represents p-value less than 1.00E-05, and ** represents p-value less than 0.001).

Zoom scan spectra of labeled and unlabeled peptide pairs were used to determine secreted protein ratios in TNF-α-treated cells versus untreated cells. Figure 4 shows example spectra of labeled and unlabeled peptide pairs of complement C3 and syndecan-4 whose secretion was significantly altered by TNF-α treatment. This differential secretion was clearly maintained in both forward and reverse SILAC experiments thus validating the data. Among the 146 proteins reported in Appendix 1, only 6 were found to be altered in their concentrations after TNF-α treatment (Figure 5). Trans-golgi network protein-2 (TGON2) was the only protein whose secretion was decreased (by a factor of 2±0.01) in TNF-α treated cells versus untreated cells while the other 5 proteins, complement C3, fibronectin, sulfhydryl oxidase 1, plasminogen activator inhibitor 1 and syndercan-4 were increased by a factor of 2.03±0.42, 1.70±0.12, 2.04±0.26, 1.92±0.16, 4.35±0.54, respectively.

**Figure 6:**
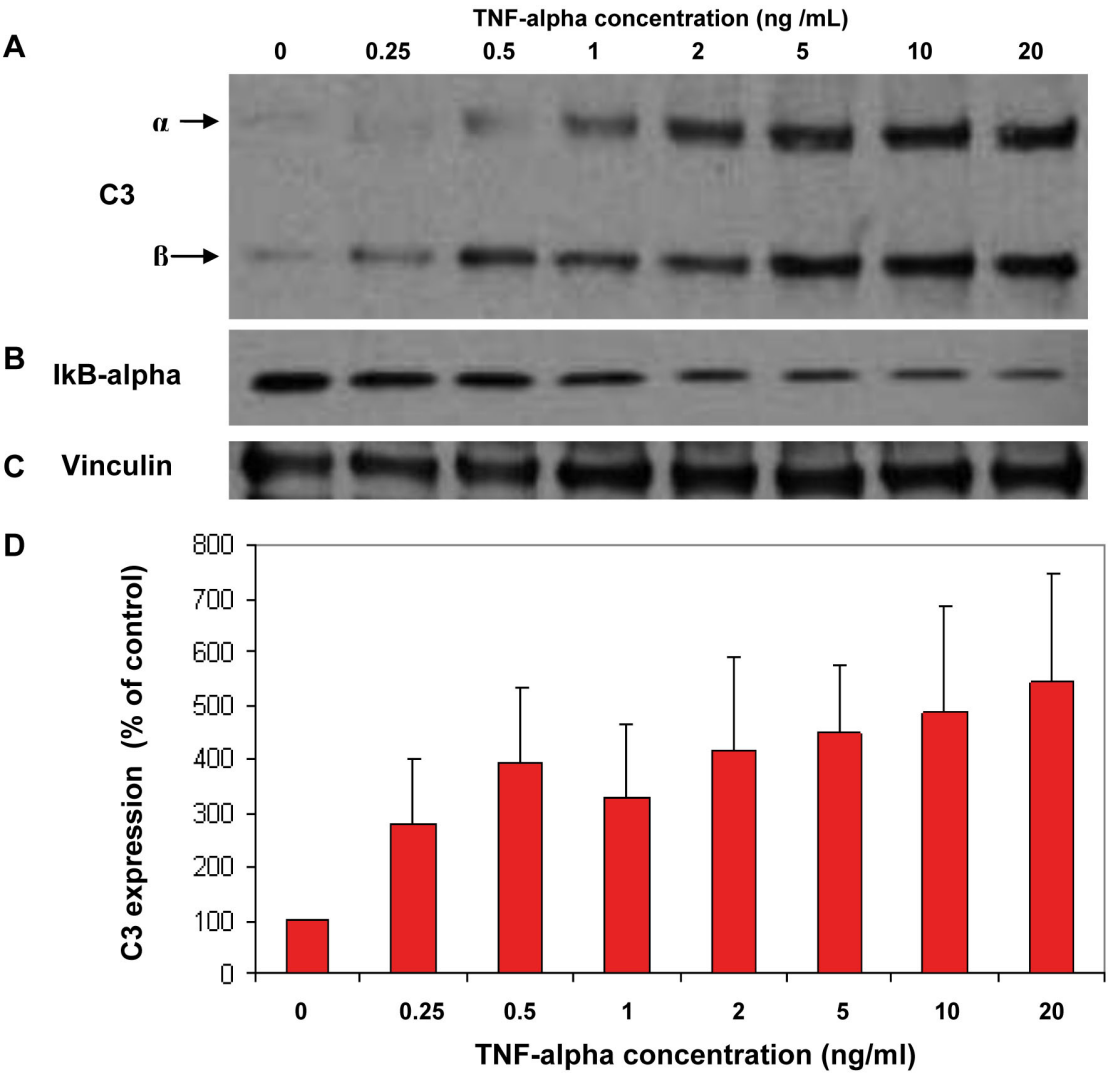
Effect of increasing concentrations of TNF-α on the secretion of Complement C3 by ARPE-19 cells. Cells were treated with increasing concentrations of TNF-α ranging from 0 to 20 ng/ml for 24 h in serum-free media. Spent media were collected, and the levels of each protein were evaluated by western blot analysis. Panel **A** shows western blot against complement C3; both C3-α and C3-β subunits are detected. Panel **B** shows western blot against cytosolic IκB-α revealing activation of NF-κB signaling pathway. **C:** western blot against vinculin, a housekeeping protein whose level remained unchanged following TNF-α treatment. Panel **D** shows bar graphs of the relative expression of complement C3 to vinculin expression in function of different TNF-α concentration. Standard deviations were obtained from 3 replicate experiments with p-values <0.05.

Because of the importance of the complement pathway in AMD, secretion of C3 was further investigated in ARPE-19 cells treated with different doses of TNF-α. Vinculin, whose abundance remained unchanged between TNF-α treated and untreated cells, was used as a loading control for data normalization while IκB-α degradation was used to monitor the TNF-α induced NF-κB pathway. As shown in Figure 6, the secretion of C3 was increased with TNF-α in dose dependant manner while I-κB significantly decreased, indicating activation of the NF-κB pathway. This experiment was repeated three times, yielding same trend of C3 expression as a function of TNF-α concentration.

## Discussion

Human ARPE-19 cells secret a variety of proteins that are involved in regulation and maintenance of the extracellular matrix, complement pathway, as well as immune and inflammatory responses. Only six proteins among the total identified proteins in ARPE-19 condition medium were found altered in their quantities following 24 h treatment with TNF-α. This response indicates that ARPE-19 cells possess a cell surface receptor for TNF-α that acts on specific gene expression via activation of the NF-κB pathway [19,33]. Indeed, this pathway was further confirmed by the observation of a TNF-α dose-dependant decrease of IκB-α, which resulted in the activation and nuclear translocation of the transcription factor NF-κB [34]. The promoter regions of the genes encoding for complement C3, plasminogen activator inhibitor, and syndercan-4 have a binding site for NF-κB as judged from the literature and as determined by TFSEARCH algorithm using 1,000 bp downstream of the promoter region. Therefore, these genes are targets for response to TNF-α treatment. Interestingly, the three other proteins altered by TNF-α (e.g., TGON2, fibronectin, and sulfhydryl oxidase 1) do not have any known binding site for NF-κB in the promoter region of their corresponding genes indicating that the regulation of these proteins by TNF-α may occur via alternative pathways or involves intermediate proteins. Previous studies have shown that TNF-α also induces secretion of a variety of chemokines (e.g., interleukin-8, growth-regulated protein-GRO, MCP-1), and cytokines (endothelin-1) in RPE cells [35–37]. Most of these studies used an immunoassay based assay or mRNA expression assays that amplify the signal of these molecules and facilitate their detection. In the present study, we were not able to detect these components using our proteomic approach. This is most likely due to their inherent limited amount and their short half life. Additionally, these proteins are generally of low molecular mass (<10 kDa) and may generate only few tryptic peptides to be detected by mass spectrometry. This shows the limitation of the proteomic approach in detecting this class of proteins and prompt for additional strategies to increase proteome coverage in the future. Nevertheless, proteomics studies allow simultaneous detection and quantification of several proteins that otherwise would have been overlooked in hypothesis-driven studies. Using a proteomics approach, we identified additional proteins whose secretion from ARPE-19 cells was influenced by TNF-α.

### Complement C3

The secretion of this protein was significantly induced by TNF-α in ARPE-19 cells. This result is in agreement with previous studies where it has been shown that cytokines regulate C3 production in different cell types including human intestinal epithelial cell line, Caco-2 [38] as well as rat glomerular endothelial cells [39]. The production of C3 in interleukin-1-treated intestinal cells was abolished by NF-κB inhibitors [38], indicating that C3 is a target gene for NF-κB transcription factor. It is most likely that a similar pathway is involved in TNF-α-treated ARPE-19. However, to the best of our knowledge, this is the first time that TNF-α has been shown to induce C3 secretion in human RPE cells. Local secretion of C3 by RPE cells could be detrimental in individuals carrying Y402H CFH variant since CFH is the physiologic inhibitor of C3 activation and protect cells from autologous injuries by complement C3 [40]. Additionally, it has been shown that monocytes, which are a major source of TNF-α, are attracted by sub-RPE deposits [41]. Therefore, in individuals with compromised CFH activity due to the Y402H mutation, this could generate a positive-feedback loop whereby the newly attracted mononcytes generate a chronic exposure of RPE cells to cytokines. In turn, this leads to increased secretion of C3 by these cells, leading to progressive immune complex deposition and autologous complement-mediated RPE injury. This hypothesis remains however to be verified in a model system using primary RPE cells with mutated and wild type CFH.

### Fibronectin and syndecan-4

Secretion of fibronectin and syndecan-4 was significantly induced in TNF-α-treated ARPE-19 cells. Syndecan-4, especially, was threefold to fivefold more abundant in the spent medium of TNF-α-treated cells versus untreated cells. These two proteins are major constituents of the extracellular matrix (ECM) and play a crucial role in cell adhesion and connectivity. While fibronectin is an extracellular protein that adheres to the cell surface to maintain their integrity and shape, syndecan-4 is a single pass transmembrane protein composed of an extracellular domain “ectodomain” bearing four heparan sulfate chains, a transmembrane domain, and a short C-terminal cytoplasmic domain.

**Figure 7:**
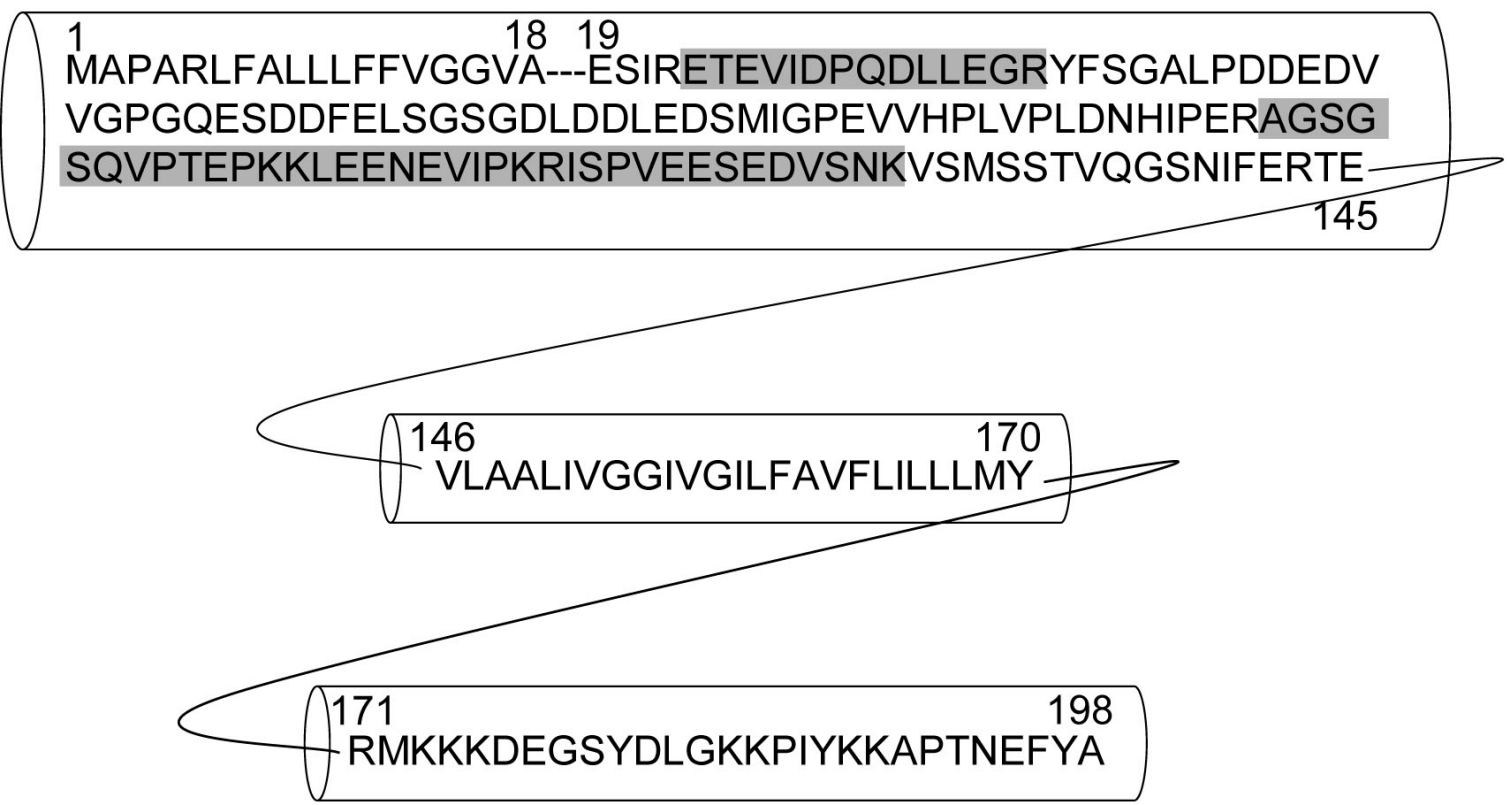
Sequence and cellular domains of human syndecan 4. The sequence from amino acid 1 to 18 represents the signal peptide and is usually cleaved out in the endoplasmic reticulum before the protein is trafficked to the cell surface. Sequence from 19 to 45 represents the extracellular domain of the protein. Sequence from 146 to 170 represents the transmembrane domain and the sequence from 171 to 198 represents the C-terminal cytoplasmic domain. Gray highlights tryptic peptides that were detected and identified by LC-MS/MS analysis. Note that all the detected peptides belonged to the extracellular domain of the protein suggesting that syndecan 4 sheds its ectodomain into the condition medium in response to TNF-α treatment.

Even though treatment of ARPE-19 with TNF-α resulted in a significant increase of fibronectin secretion neither the literature nor software prediction support the involvement of the NF-κB pathway in this process, suggesting that this protein might be indirectly upregulated. Indeed, fibronectin was identified in SDS–PAGE around 100 to 250 kDa indicating that the protein was fragmented [42]. Upregulation of fibronectin fragments might be due to proteases involved in extracellular matrix degradation such as the serine protease HtrA1 [41]. Interestingly, mutation in the promoter region of the gene encoding for HTrA1 was recently found to be strongly associated with AMD pathology [9–11]. Nevertheless, the exact mechanism by which this single point mutation leads to drusen formation and macular degeneration remains to be further investigated. Although the molecular mass of syndecan-4 core protein is around 21 kDa, this protein was detected at around 250 kDa in our SDS–PAGE. This abnormal SDS–PAGE migration is most probably due to the O-linked glycosaminglycan chains. This hypothesis is in agreement with previous studies where it has been shown that intact syndecan migrates at higher molecular mass in the gel than does heparinase-treated syndecan [43]. All the peptides identifying this protein in our study belonged to the ectodomain sequence (Figure 7) suggesting that the N-terminal part of the protein is shed into the spent medium of TNF-α treated ARPE-19 cells. Previous study has shown that TNF-α induces the overall expression of syndecan-4 in endothelial cells via NF-κB activation [44]. Other studies have shown that syndecan-4 shedding is mediated by matrix metalloproteinase-9 (MMP9) and is dependent of NF-κB signaling [45]. In our proteomic profiling data we did detect 2 peptides for MMP9 and these were found to be more abundant in TNF-α-treated than untreated cells (data not shown). Unfortunately, due to the inherent low amount of this protein in the spent medium of ARPE-19, we were not able to detect these same peptides in our reverse experiment. What remains to be examined is whether the increased amount of syndecan-4 seen in the spent medium of TNF-α-treated ARPE-19 cells was due to induction of its expression via NF-κB activation or to the increased shedding of its ectodomain. In either case, shedding of this from the surface of ARPE-19 cells exposed to TNF-α could be a protective mechanism against TNF-α cytotoxicity, and this may explain the resistance of these cells to TNF-α-induced apoptosis [32]. Indeed, it has been documented that the TNF-α signaling pathway synergizes with the binding of matricellular protein CCN1/CYR61 to syndecan-4 ectodomain and triggers a cascade leading to apoptosis [46]. Nevertheless, the contribution of this shedding to AMD pathogenesis remains to be carefully examined even though some studies demonstrated that drusen contained some glycosaminoglycan and heparan sulfate-conjugated proteoglycans [47,48]. Also, at present it is uncertain whether CFH, an important factor in AMD pathology, binds to heparan sulfate of syndecans and whether shedding of syndecan will make cell more vulnerable to increased complement C3 secretion. More studies on the interactions between cell surface proteoglycan and CFH (both the Y402H variant and the wild type) are needed.

### Plasminogen activator inhibitor-1

Based on the spectral count of total peptides generated per protein, plasminogen activator inhibitor-1 (PAI-1) appears as one of the highly abundant proteins in the spent medium of ARPE-19 cells (29 peptides and 1474 spectral count were detected for this protein even under basal conditions). The secretion of PAI-1 was further increased by twofold in TNF-α-treated ARPE-19 cells versus untreated cells. Previous studies have shown that TNF-α readily induces PAI-1 in different cell types [49–52]. PAI-1 is a physiologic inhibitor of plasminogen activator and plays an important role in the regulation of the extracellular matrix. Its expression is increased in several fibrotic diseases and regulated by various cytokines, growth factors, and chemical/physical agents [53]. RPE cells secrete both urokinase plasminogen activator and its inhibitor PAI-1 [54] and the balance between these two components likely plays a crucial role in maintaining the extracellular environment of RPE cells including regulation of angiogenesis [55–58]. Interestingly, PAI-1 was found to be more abundant in the serum of patients with early or advanced AMD than of healthy individuals, suggesting its potential implication in AMD pathogenesis [59].

### Sulfhydryl oxidase 1

A protein also known as quiescin Q6 (QSOX), sulfhydryl oxidase 1 has 2 known isoforms generated by alternative splicing. Isoform 1, a single pass membrane protein, can be found in the endoplasmic reticulum and Golgi apparatus while isoform 2, missing the C-terminal domain of isoform 1, is mainly found in the extracellular space [60]. All the 17 peptides identified for this protein in our study belonged to isoform 2, consistent with a secreted form. In other words, we did not detect any peptide sequence belonging to the C-terminal domain of isoform 1. QSOX protein utilizes a thioredoxin domain and a small flavin adenine dinucleotide (FAD)-binding domain to form disulfide bonds in proteins and peptides especially in proteins destined for secretion [60]. QSOX is typically found in the cell along the secretory machinery and in the extracellular space. However, little is known about the secreted form and its significance. Whether this secreted form has a role in disulfide bond formation of proteins in the extracellular space or is merely carried along with proteins during their secretion remain to be determined. Nevertheless and to the best of our knowledge, this is the first demonstration that TNF-α induces secretion of QSOX in human RPE cells despite the lack of a binding site for activated NF-κB promoter region of its gene. It is believed that this protein may play a crucial role in ECM remodeling [61] which might be relevant in diseases involving ECM deposition such as AMD, Alzheimer and fibrosis.

### Trans-golgi network protein 2

**Figure 8:**
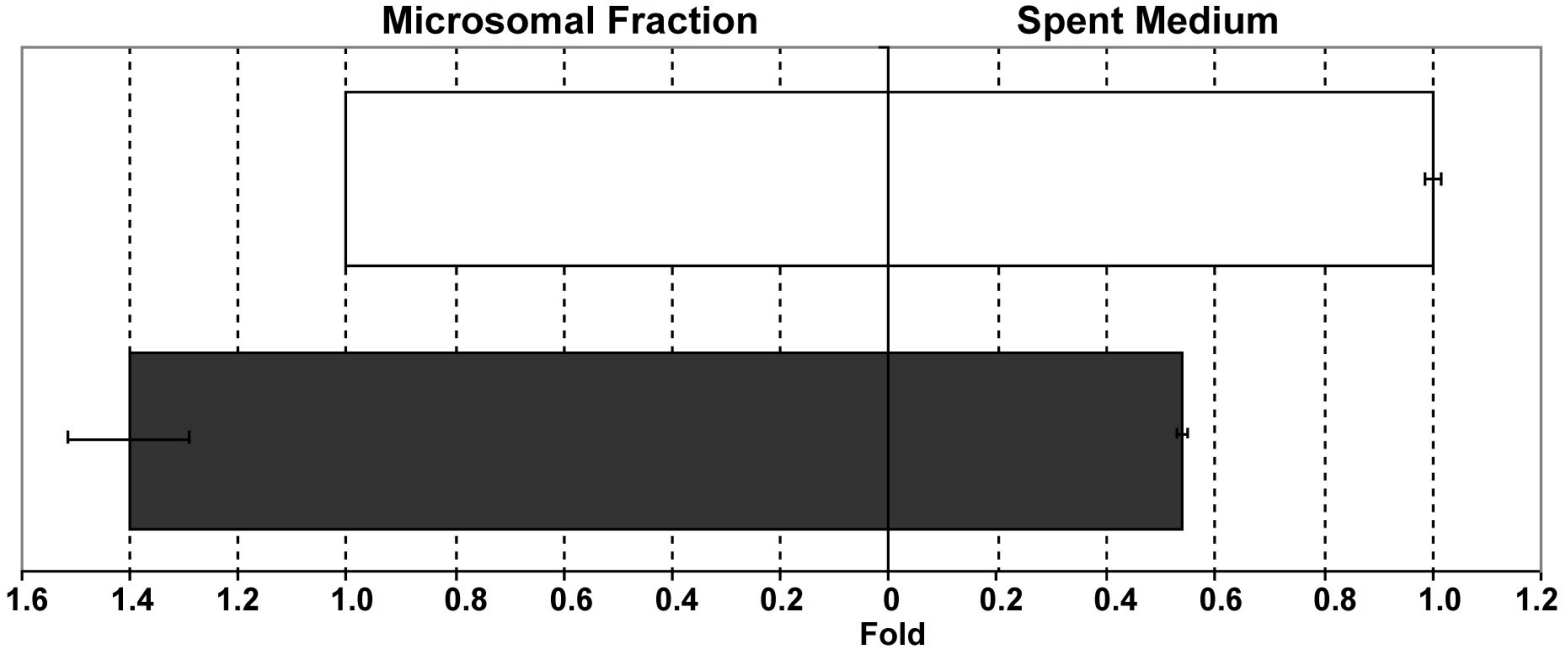
Effect of TNF-α on the subcellular distribution of the trans-golgi network protein-2. Trans-golgi network protein-2 was found to decrease in the extracellular space and increase in the microsomal fraction in TNF-α-treated ARPE-19 cells (dark bars) when compared to untreated ARPE-19 cells (clear bars). This strongly suggests that TNF-α influences trans-golgi network protein-2 trafficking in ARPE-19 cells.

TGON2 was the only protein that was found to be decreased in the spent medium of TNF-α-treated versus untreated ARPE-19 cells. While TGON2 was decreased in the extracellular space of treated cells, its amount was actually increased in the microsomal fraction of this same set of cells (Figure 8), indicating that the changes were due to retention of this protein rather than inhibition of its expression. Indeed, TGON2 is involved in regulating membrane traffic to and from the trans-Golgi network. RPE cells express both TNF receptor-associated factor-1 and 2 (TRAF-1 and TRAF-2) at their surface [33]. The decreased amount of TGON2 at the surface of TNF-α-treated cells could be associated with the internalization of TNF-α/ TNF-α receptor (TNFR) complex to modulate TNF-signaling [62,63]. Indeed, it has been shown that TNFR can be localized both at the cell surface and to the Golgi apparatus of endothelial cells, and that TNF-α binding modulates its trafficking [64].

Overall this study showed that TNF-α modulate the RPE secretion of a variety of proteins that play a major role in extracellular matrix remodeling, complement network and angiogenesis which might be relevant to AMD pathogenesis. Although preliminary, this study demonstrated that TNF-α induced secretion of specific proteins in ARPE19 cells. Even though these cells are not an ideal RPE model in respect to primary cultures, they were used as proof of principle to demonstrate that several secreted proteins can be qualitatively and quantitatively monitored in TNF-α-treated versus untreated cells. These proteins set may serve as a catalog for future targeted studies that use human primary RPE cells or human retinal cross-sections.

## Appendix 1. List of proteins found in the condition media of ARPE-19 cell cultures

Serum free condition media were collected from 24 h ARPE-19 cell cultures. The protein content in these media were concentrated and separated by SDS–PAGE. Gel bands where excised, digested with trypsin and the resulting peptides analyzed by LC-MS/MS as described in the method. UniProt ID column shows accession number for each identified protein. Peptide count column shows the number of tryptic peptides detected and identified for each protein. The last column indicates the subcellular localization of each protein. Each of these proteins were identified by at least two peptides and validated by detection of both labeled and unlabeled peptide pairs. To access the data, click or select the words “Appendix 1.” This will initiate the download of a compressed (pdf) archive that contains the file.

## Appendix 2. Additional proteins identified by a single peptide pair in the spent medium of ARPE-19 cells

Additional proteins detected in the spent media of ARPE-19 cell cultures were represented by a single tryptic peptide. The identity of this single peptide was validated by the presence of its labeled homolog peptide. UniProt ID column indicates the accession number of each of these proteins. Peptide count column indicates the number of peptides identified for each of these proteins. The Scan count column indicates the number of times this peptide was detected and analyzed by the mass spectrometer. The higher the number the more abundant is the peptide. The last column indicates the subcellular location of each of these proteins. To access the data, click or select the words “Appendix 2.” This will initiate the download of a compressed (pdf) archive that contains the file.
